# The Cystic Fibrosis Impact Questionnaire: qualitative development and cognitive evaluation of a new patient-reported outcome instrument to assess the life impacts of cystic fibrosis

**DOI:** 10.1186/s41687-020-00199-5

**Published:** 2020-05-13

**Authors:** Kelly P. McCarrier, Mariam Hassan, Paul Hodgkins, Ellison Suthoff, Lisa J. McGarry, Mona L. Martin

**Affiliations:** 1 Pharmerit International, 4350 East-West Highway, Suite 1100, Bethesda, MD 20814 USA; 2 Insmed Pharmaceuticals, Bridgewater, NJ USA; 3 Vertex Pharmaceuticals, Boston, MA USA; 4 Sage Therapeutics, Boston, MA USA; 5 Evidera/PPD, Seattle, WA USA

**Keywords:** Cystic fibrosis, Patient-reported outcome, Content validity, Qualitative research, Scale development

## Abstract

**Background:**

Patients with cystic fibrosis (CF) experience significant disease burden, including progressive pulmonary decline and reduced survival. This multicenter qualitative study was conducted to develop a new patient-reported outcome (PRO) measure to assess the impact of CF on patients’ quality of life: the Cystic Fibrosis Impact Questionnaire (CF-IQ).

Semi-structured qualitative concept elicitation (CE) interviews with patients and caregivers documented CF-related symptoms, impacts, and treatment experiences. Coded interview data were considered alongside existing PROs, published literature, and expert opinion to develop an initial scale. Three rounds of cognitive interviews evaluated respondent comprehension and facilitated refinement of the CF-IQ.

**Results:**

Adult (*N* = 20) and pediatric (*N* = 22) patients with CF and their parents/caregivers (*N* = 22) completed CE interviews at 7 US clinics. The sample included patients aged 6–58 years, 57% females, and represented a broad range of disease severity (forced expiratory volume in 1 s range: 22%–127% predicted). Interviews identified 59 unique CF-related impact concepts in domains, including activity limitations (physical, social, leisure), functional limitations (school, work), vulnerability/lack of control, emotional impact, treatment burden, and future outlook. Concept saturation was achieved, and a draft questionnaire was developed. Findings from the cognitive interviews (*n* = 18) confirmed that instructions, items, and response scales were relevant and clear, and interpreted as intended by patients.

**Conclusion:**

The CF-IQ is a 40-item novel PRO scale assessing a comprehensive set of patient-relevant concepts to characterize the multifaceted nature of CF. Qualitative interview data support the content validity of the CF-IQ, which is currently undergoing additional psychometric evaluation in patients with CF.

## Background

Cystic fibrosis (CF) is a rare, autosomal recessive disease that affects an estimated 30,000 individuals in the US and more than 70,000 people worldwide; approximately 1000 new cases are diagnosed in the US each year [1]. CF is associated with serious, chronically debilitating morbidities and premature mortality. In 2017, the median age at death for patients with CF was 30.7 years, while the predicted median age of survival for patients born in 2017 was 46.2 years, reflecting increased expected longevity associated with recent advancements in CF care [2]. CF affects multiple organs (lungs, pancreas, sweat glands, and intestinal, biliary, and reproductive tracts) [3], and pulmonary disease is the primary cause of morbidity and mortality [4]. Patients with CF typically experience progressive loss of lung function, often resulting in respiratory failure and death [3, 5].

Patients with CF experience substantial decrements in health-related quality of life (HRQoL) arising from symptoms, functional limitations, high levels of treatment burden, and other complicating aspects of the condition [6], such as progressively declining lung function and repeated pulmonary exacerbations [7]. The treatment burden of CF is significant, with most patients spending more than 2 h per day on routine maintenance therapies, which involve taking multiple oral and inhaled medications, and chest physical therapy [8, 9].

Beyond the direct impact of symptoms and treatment burden on HRQoL, children and adolescents report feelings of vulnerability, loss of independence and opportunities, stigmatization, concern about getting sick, social isolation, lack of control, and resentment of long-term treatment. Children also report fear of injections and fatigue from prescribed physical exercise and describe their CF treatment as intensive and invasive [10]. Longitudinal studies in adults with CF have shown steady decreases in social functioning over time [6, 7]. Patients report interferences with activities of daily living, such as work and school, and treatment burden can further increase with added therapies, clinic visits, and hospitalizations resulting from acute infections and exacerbations [11].

Due to the substantial influence of CF on the quality of life of patients, rigorous assessment of these impacts is critical in understanding the potential benefits of new and emerging CF therapies. Because CF is a multisystemic disease, prior to the introduction of CF transmembrane conductance regulator (CFTR) modulators, therapy included symptomatic treatment and management of pulmonary exacerbations and other complications by controlling airway infections and inflammation, mobilizing secretions to reduce airway obstruction, and correcting gastrointestinal symptoms and nutritional deficits caused by pancreatic insufficiency [12–15]. The introduction of CFTR modulators allows treatment to advance from symptom reduction to targeting the underlying cause of the disease. With changes in the treatment landscape, there is an emerging place for patient-reported outcome (PRO) instruments for CF that capture the broad experience of the patients, which will be useful in understanding the totality of the benefits of the newer therapies.

The few CF-specific PRO instruments currently available focus largely on symptom assessment and were developed before the introduction of CFTR modulators as a therapeutic option, and prior to current PRO development guidelines emphasizing the importance of direct patient input to determine the concepts that are most relevant and important to the target patient population [16–18]. Three previously developed disease-specific PRO instruments for assessment of CF concepts are the Cystic Fibrosis Quality of Life (CFQoL) assessment [19], the Cystic Fibrosis Questionnaire-Revised (CFQ-R) [20–22], and the Questions on Life Satisfaction for Adolescents and Adults with Cystic Fibrosis (FLZ^M^-CF) [23].

The CFQoL is an instrument that consists of 52 items across 9 domains: physical functioning, social functioning, treatment issues, chest symptoms, emotional responses, concerns for future, interpersonal relationships, body image, and career concerns [19]. The FLZ^M^-CF is an 18-item CF-specific add-on module to the parent life satisfaction measure that includes items assessing satisfaction with symptoms (i.e. breathing difficulty/coughing, abdominal pain, digestive trouble, sleep difficulty) and other elements of overall disease burden (i.e. integration of therapy into daily routine, being needed by others, having your situation understood by others) [23]. Although the development of both instruments did include limited qualitative patient input, it is unclear from the available documentation whether the CFQoL or FLZ^M^-CF were constructed consistently with current scientific standards for evidence of content validity in PRO development [16, 19].

The CFQ-R consists of 44 items over 9 HRQoL domains (physical, role/school, vitality, emotion, social, body image, eating, treatment burden, health perceptions) and 3 symptom scales for weight, respiratory function, and digestion [21]. The CFQ-R has demonstrated the ability to detect important differences in patient QoL in both the clinical trial and real-world settings [24–26] and is likely to remain the gold standard for clinical drug development. However, like the CFQoL and FLZ^M^-CF, the CFQ-R was developed prior to the introduction of CFTR modulator-based therapies, and while this instument provides important information on quality of life, it may not be fully optimized to capture a patient’s full experience of life with CF and the impact of new therapies.

To ensure incorporation of the most relevant concepts that extend beyond the measurement of CF symptoms, the present study utilized qualitative concept elicitation and cognitive interview methods to assess the HRQoL impacts on patients with CF and to develop the Cystic Fibrosis Impact Questionnaire (CF-IQ). Our goal in developing the CF-IQ is to complement and build on the available PRO instruments by assessing a comprehensive set of patient-relevant and important concepts to characterize the multifaceted nature of disease burden in CF as experienced by patients within the modern therapeutic environment, including those receiving therapy with CFTR modulators.

## Methods

### Study design

PRO instrument development employed qualitative data collection and analysis techniques consistent with scientific best practices [16–18].

A review of the literature, including evaluation of existing PRO instruments used in CF clinical studies, was conducted to inform instrument development. The review identified 45 different PRO instruments used in published CF studies during the 15-year review period. Only four of the identified instruments were developed as CF-specific assessments, with three of those (the CFQoL, FLZ^M^-CF, and CFQ-R, as detailed above) including coverage of concepts related to the impact of CF on daily life and treatment-related experiences, as opposed to assessment limited to CF symptoms.

The review was followed by qualitative interviews with clinicians and nurses providing care for patients with CF. The study team used input from CF specialists and instrument development experts, as well as the findings from the literature review and clinician interviews, to inform protocol design and to develop semi-structured interview guides for use in qualitative data collection with patients with CF and their caregivers.

**Fig. 1:**
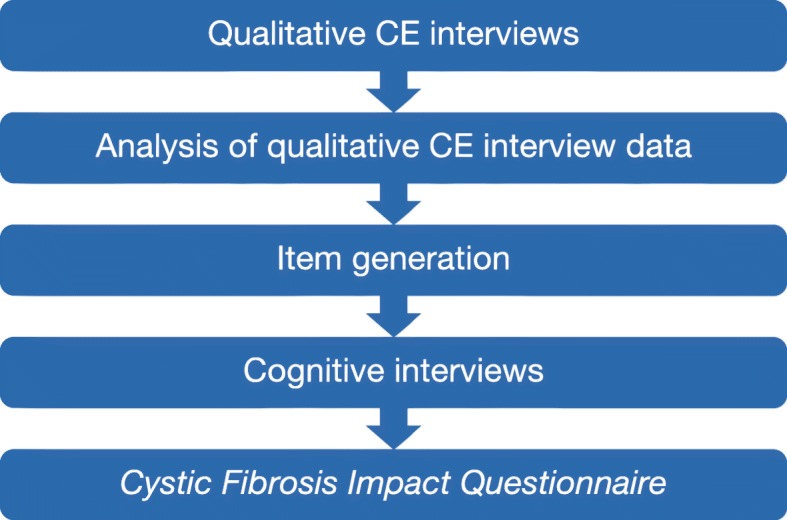
Process flow chart for development of the Cystic Fibrosis Impact Questionnaire. CE concept elicitation

Following development of interview guides, in-person qualitative concept elicitation interviews were conducted with pediatric, adolescent, and adult patients with CF to identify symptoms, symptom-related impacts, and treatment experiences relevant and important to patients. Primary caregivers of participating children aged 6–17 years were also interviewed. All interviews were audio-recorded and transcribed for concept coding. Figure 1 provides an overview of the key tasks undertaken in PRO development.

### Study population

Eight CF treatment centers across the US recruited patients from their ongoing caseloads to participate in the qualitative interviews. Seven sites enrolled patients for the initial concept elicitation interviews and 4 sites (3 remaining from the concept elicitation phase plus 1 additional clinic) enrolled patients during the cognitive interview phase. A central institutional review board (IRB), Quorum Review IRB (Seattle, WA, USA), approved the study protocol and materials, as did local IRBs where required. All patients (or parents/legal guardians of patients aged < 18 years) provided written informed consent prior to initiation of any study-related procedures. No changes to the participants’ existing care were made, and no clinical interventions were initiated as part of the study protocol.

A purposive, non-probability sample of approximately 40–50 patients with CF was planned for the concept elicitation phase. Eligible patients had a physician-confirmed diagnosis of CF, defined as a sweat chloride value ≥60 mmol/L by quantitative pilocarpine iontophoresis, or 2 documented CF-causing mutations, and either chronic sinopulmonary disease or gastro-intestinal/nutritional abnormalities. Recruitment quotas within 5 age groups (6–8, 9–11, 12–17, 18–24, and ≥ 25 years) were used to capture age-related differences over the course of the disease, as well as in type and intensity of life activities. To reflect the range of comorbidities seen in patients with CF, additional recruitment targets were used to guide the inclusion of patients in 2 key clinical subgroups: 1) patients with comorbid CF-related diabetes were limited to ≤33% of the overall sample; and 2) patients with more severe CF presentations (defined as a history of ≥2 hospitalizations in the previous year for patients aged ≥18 years and ≥ 1 hospitalization in the prior year for patients aged < 18 years) were targeted to represent approximately 33% of the overall sample. Recruitment targets were set for approximately 50% of the sample to be composed of patients receiving CFTR modulator therapy at the time of interview.

During the enrollment process, patients completed a questionnaire to provide demographic characteristics (age, sex, ethnicity, race, educational attainment, employment status) and ratings of their overall health compared with others and the overall severity of their CF symptoms.

### Qualitative concept elicitation interviews

Individual concept elicitation interviews were conducted to gather information about key symptoms and disease-related life impacts experienced by patients with CF, as well as functional changes in daily living following treatment. All interviews lasted approximately 60–90 min and used a semi-structured interview guide, starting with general, open-ended questions and a day reconstruction exercise, and continuing with a structured probing system to gather more detailed information about participants’ symptoms, disease impacts, and treatment-related experiences. The probe system was informed by clinician interviews and a systematic review of existing CF literature. Separate, age-appropriate interview guides were used with children (aged 6–11 years), adolescents/adult patients (aged ≥12 years), and the caregivers of participating patients aged < 18 years. Interviewers followed each institution’s infection control protocols (i.e. mask, gloves, gown procedures) during the patient interviews. Young children were interviewed with a parent or caregiver present, if desired, but they provided their own responses to interview questions. In most cases, the caregiver interviews were conducted separately, without the child present in the room at the time the caregiver provided his/her responses.

### Analyses

All interviews were audio-recorded and transcribed. Transcript coding was conducted using ATLAS.ti software (ATLAS.ti Scientific Software Development GmbH, Berlin, Germany) [27], and coded expressions were grouped by similarity of content for analysis. Coded transcript data were evaluated for evidence of saturation of concept (defined as the point at which no new concept-level information was forthcoming from the study sample) to confirm the adequacy of the sample size and robustness of the set of concepts elicited. To detect the point of concept saturation, a summary saturation table was used to track the occurrence of novel impact concepts expressed within chronologically ordered sets of interviews. Consistency and reproducibility of concept coding were assessed via evaluation of inter-rater agreement (IRA).

### Item generation

The development team met with CF clinical experts and clinical outcome assessment measurement experts to review results of the concept elicitation interviews and identify the concepts most appropriate to include in a CF-impact PRO. The hypothesized relationships between the selected concepts and domains of the draft instrument were modeled in a preliminary conceptual framework. For each selected concept, transcript data were used to develop draft PRO item text (i.e. question and scale) reflecting the choice of words and descriptions used by patients to articulate the concept.

### Cognitive interviews

Following the development of the preliminary draft instrument, cognitive interviews were conducted to evaluate concept relevance and understandability, the structure of the draft items and response scales, and the appropriateness of the recall period. Interviews were conducted in 3 iterative waves; the development team reviewed the findings from each wave and implemented refinements to the draft instrument before each subsequent set of interviews. The preliminary conceptual framework for the draft instrument was adjusted based on refinements made during the cognitive interviews.

Cognitive interview participants were selected through the same recruitment process used for qualitative interviews. Cognitive interviews used a semi-structured interview guide, which included broad questions to assess the comprehensiveness and relevance of the concept coverage of the CF-IQ, as well as a “think-aloud” process to ensure the instructions, items, and response options were interpreted as intended for each item of the CF-IQ [18, 28]. When possible, questions were reframed in the terminology used by respondents to aid comprehension.

## Results

### Characteristics of study sample

A total of 42 qualitative patient interviews were conducted with English-speaking adult (*N* = 20) and child (*N* = 22) participants, as well as 22 adult caregivers (Table 1). The population was well distributed across the 5 quota-targeted age groups and differing levels of education. Twenty-four (57%) patients were female. Twelve of the 20 adult patients were employed either full-time or part-time, 3 were students, and 5 were not working outside of the home at the time of the interview. Seventeen (41%) of the concept elicitation interview participants (9 adult and 8 pediatric patients) were receiving treatment with a CFTR modulator, and 13 participants (7 children/adolescents and 6 adults; 30.9% of the overall sample of 42) had experienced ≥2 hospitalizations in the previous year.

**Table 1:** Participant demographic and clinical characteristics

Characteristic	Patient concept elicitation participants (*N* = 42)	Caregiver concept elicitation participants (*N* = 22)	Cognitive interview participants (*N* = 18)
Age, years, mean (median; range)	20.9 (17.4; 6.7–58.2)	42.6 (41.5; 33.2–60.3)	20.6 (19.1; 9.0–33.0)
Age quota group, years, n (%)			
6–8	6 (14.3)	N/A	0
9–11	6 (14.3)	N/A	1 (5.6)
12–17	10 (23.8)	N/A	6 (33.3)
18–24	8 (19.0)	N/A	6 (33.3)
≥ 25 or older	12 (28. 6)	N/A	5 (27.8)
Sex, n (%)			
Male	18 (42.9)	5 (22.7)	11 (61.1)
Female	24 (57.1)	17 (77.3)	7 (38.9)
Hispanic, Latino, or Spanish origin, n (%)			
Not Hispanic or Latino	38 (90.5)	20 (90.9)	18 (100.0)
Hispanic or Latino	4 (9.5)	2 (9.1)	0
Race, n (%)			
Black or African American	2 (4.8)	1 (4.5)	0
Native Hawaiian or other Pacific Islander	1 (2.4)	0	0
White	37 (88.1)	21 (95.5)	16 (88.9)
Other	2 (4.8)	0	2 (11.1)
Highest education level completed, n (%)			
Less than high school	18 (42.9)	0	3 (16.7)
High school	9 (21.4)	3 (13.6)	8 (44.4)
College	4 (9.5)	6 (27.3)	4 (22.2)
Bachelor’s degree	6 (14.3)	13 (59.1)	2 (11.1)
Graduate or professional school	5 (11.9)	0	1 (5.6)
Employment status, n (%)			
Employed full-time for wages	7 (35.0)	N/A	1 (5.6)
Employed part-time for wages	5 (25.0)	N/A	6 (33.3)
Out of work for < 1 year	0	N/A	1 (5.6)
Out of work for > 1 year	1 (5.0)	N/A	2 (11.1)
Homemaker	1 (5.0)	N/A	0
Student	3 (15.0)	N/A	1 (5.6)
Retired	1 (5.0)	N/A	0
Unable to work	2 (10.0)	N/A	0
Not asked^a^	22		7
Patients’ self-rated overall health (compared with their peers), n (%)			
Poor	2 (6.7)	N/A	1 (5.6)
Fair	8 (26.7)	N/A	3 (16.7)
Good	9 (30.0)	N/A	9 (50.0)
Very good	9 (30.0)	N/A	2 (11.1)
Excellent	2 (6.7)	N/A	2 (11.1)
Not asked^b^	12		1
Patients’ self-rated severity of CF symptoms, n (%)			
No symptoms	0	N/A	1 (5.6)
Mild	10 (33.3)	N/A	5 (27.8)
Moderate	15 (50.0)	N/A	11 (61.1)
Severe	4 (13.3)	N/A	0
Very severe	1 (3.3)	N/A	0
Not asked^b^	12		1
CF-related hospitalizations in the past year, mean (median; range)	0.9 (0; 0–6)	N/A	0.72 (0; 0–2)
Duration of stay of most recent CF-related hospitalization, days (median; range)	10.4 (11.0; 2–19)	N/A	6.2 (4.0; 2–18)
Mean most recent ppFEV_1_ value	80.8 (85.0; 22–127)	N/A	64.6 (61.5; 8–115)
Comorbid conditions reported in ≥5% of participants, n (%)			
Bronchitis	2 (4.8)	N/A	3 (16.7)
Asthma	8 (19.0)	N/A	7 (38.9)
Recurring infections	13 (31.0)	N/A	1 (5.6)
CF-related diabetes	4 (9.5)	N/A	4 (22.2)
Pancreatic insufficiency	30 (71.4)	N/A	17 (94.4)
Sinus disease	13 (31.0)	N/A	9 (50.0)
GERD	5 (11.9)	N/A	5 (27.8)
Malabsorption	11 (26.2)	N/A	9 (50.0)
Depression	2 (4.8)	N/A	3 (16.7)
Anxiety	3 (7.1)	N/A	1 (5.6)
Patient is receiving CFTR modulator therapy, n (%)			
Yes	17 (40.5)	N/A	6 (33.3)
No	25 (59.5)	N/A	12 (66.6)

CF cystic fibrosis; *CFTR* CF transmembrane conductance regulator; *GERD* gastroesophageal reflux disease; *N/A* not available (variable was not collected or not applicable to the respondent); *ppFEV*_*1*_ percent predicted forced expiratory volume in 1 s

^a^Item asked only of adult patients aged ≥18 years (*n* = 20)

^b^Item asked only of patients aged ≥12 years (*n* = 30)

Patients were mostly evenly distributed between fair, good, and very good overall health, with very few extreme-level ratings (poor or excellent) (Table 1). All 30 patients aged ≥12 years had symptoms associated with CF. Although there were some ratings of severe and very severe (5 of the 30 patients), most patients rated symptom severity as mild or moderate.

### Qualitative results

Analysis of the concept elicitation interview transcripts resulted in more than 4000 coded expressions about CF symptom and impact experiences. The objective of the PRO development process was to construct an assessment of the impacts of CF (rather than its symptoms). Although the coded symptom concepts were used to illuminate the context of the patients’ downstream impact experiences, the subsequent steps focused on evaluating and selecting impact concepts for PRO measurement.

To support instrument development, coded expressions of CF impact were organized into 59 impact concepts for potential inclusion in the PRO scale. Four dual-coded transcripts were assessed for IRA, with 90.2%–95.7% agreement observed between 2 coders on concept codes assigned to text segments. With approximately 8227 words of narrative text per transcript and 4005 assignable codes, the results suggest high IRA.

Saturation of impact concepts was achieved within the first 35 of the 42 coded transcripts. No new concepts emerged in subsequent transcripts, suggesting that additional interviews were unlikely to result in additional concepts being identified, and that the sample of 42 interviews was adequate to achieve completeness of concepts from this study population (Table 2).

**Table 2:** Concept saturation: identification of novel CF impact concepts by transcript group

Transcript group (42 total interview transcripts, organized chronologically)	Concepts first coded in group, n (%)	New impact concepts identified by transcript group^a^
Group 1 (*n* = 7 transcripts)	39 (66.1)	Amount of Time for Treatment, Needing Additional Treatments, Increased Doctor’s Visits, Worry About Increased Hospitalization, Missed Opportunities due to Treatment, Resentment over Treatment, Difficulty Climbing Stairs, Difficulty Running, Limitations to Exercise/Sports, Limitations to Personal Care, Difficulty Talking, Limitations to Physical Activities in General, Feeling Weak/Lack Physical Strength, Anger, Anxiety, Distress, Embarrassment/ Self-Conscious, Fear, Frustration, Feeling Overwhelmed, Sadness, Stress, Worry, Poor Emotional Health in General, Lack of Awareness from Others, Lifestyle/Leisure Restrictions, Limited Play Opportunities, Social Isolation, Lower Productivity, School Absences, Unable to Work, Limited Goals, Uncomfortable Starting New Activities, Limited Ability to Plan, Feels Different from Others, Cost of Treatment/Care, Difficulty Falling Asleep, Difficulty Staying Asleep, Reduced Sleep Quality
Group 2 (*n* = 7 transcripts)	18 (30.5)	Difficulty Walking, Need to Rest More, Limitations to Housework/Chores, Guilt, Irritability/Moodiness, Symptoms of Depression, Vulnerability, Altered Relationships with Friends, Missed Social Activities, Work Absences, Poor Future Outlook, Feels Stigmatized, Fighting for Normalcy, Lack of Control, Overall QoL, Altered Relationships with Family, Caregiver Burden, Impact on Family Unit
Group 3 (*n* = 7 transcripts)	1 (1.7)	Cost of Inability to Work
Group 4 (*n* = 7 transcripts)	0 (0.0)	None
Group 5 (*n* = 7 transcripts)	1 (1.7)	Dating Difficulties
Group 6 (*n* = 7 transcripts)	0 (0.0)	None

CF cystic fibrosis; *QoL* quality of life

^a^First appearance of each concept. Saturation is confirmed when no new concepts arise after the earlier set of interviews

### Item generation

Following the concept elicitation phase, the development team reviewed key findings from the qualitative data, literature review, and clinical expert input to identify relevant CF impact concepts targeted for inclusion in a PRO instrument. To determine the most patient-relevant and clinically meaningful concepts for selection, the following parameters were considered: frequency of expression, total number of expressions, spontaneous nature of expressed concepts, patient ratings of difficulty coping with each impact, and clinical expertise.

This process reduced 59 coded concepts to 42 targeted concepts for inclusion in the draft CF-IQ instrument. Concepts that exhibited low prevalence among interview participants, or those that were overly general or redundant were removed.

### Cognitive interviews

A total of 18 patients participated in 3 waves of cognitive interviews (Table 1), during which patient feedback led to revisions to the wording and/or response options of several items, and the removal of 2 items. Cognitive interview data provided evidence that the revised instrument was comprehensive, relevant to the CF experience, readily understandable, and easy to complete. Patient input confirmed the appropriateness of the item structure, response scale, and recall period.

### The Cystic Fibrosis Impact Questionnaire

**Fig. 2:**
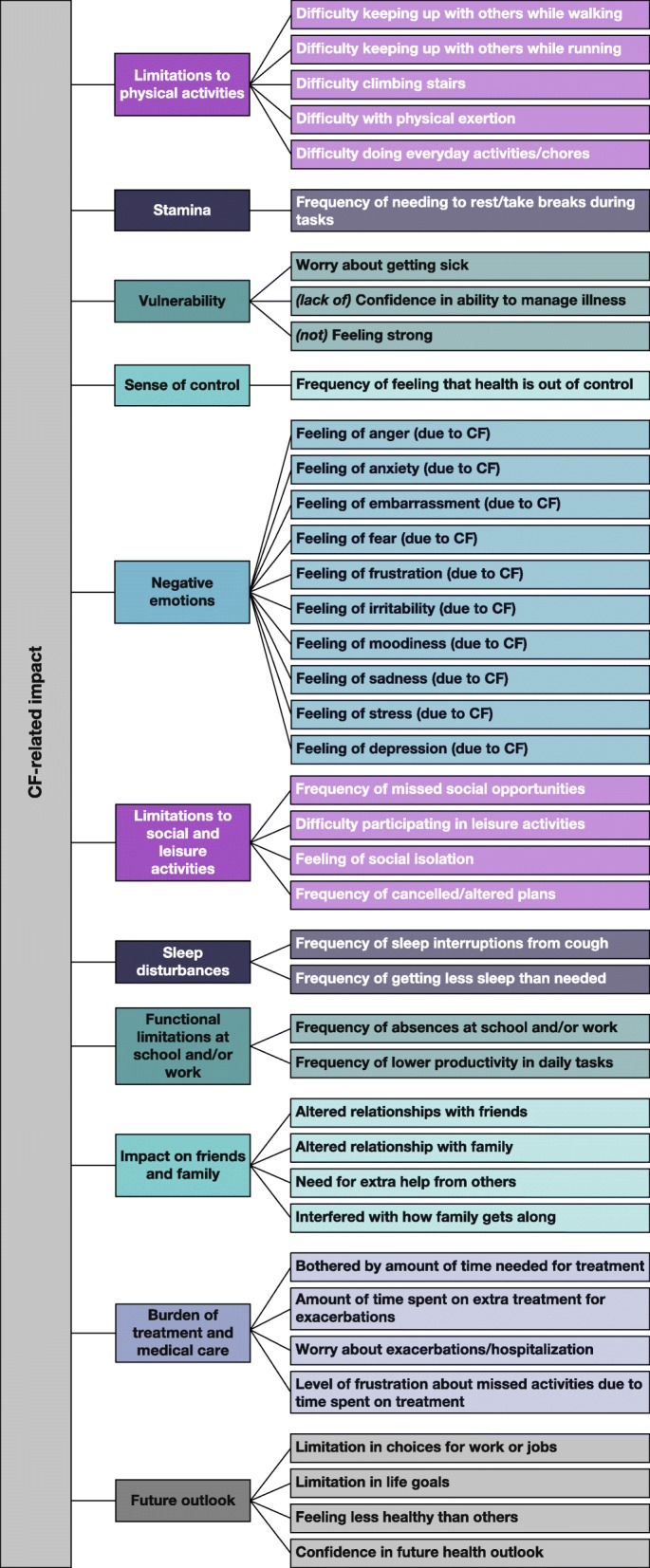
Conceptual framework for the Cystic Fibrosis Impact Questionnaire. CF cystic fibrosis

The newly created CF-IQ is a 40-item PRO questionnaire that measures each impact concept (Fig. 2) using a 5- or 7-point verbal rating scale. Thirty-two items focus on severity or intensity, with a rating scale from “no [impact]” to “extreme [impact]”. The remaining 8 items focus on frequency or amount of time an impact was experienced, and employ a rating scale from “never” to “always”. Thirty-six CF-IQ items utilize a 7-day retrospective recall period to assess the impact of CF, whereas the remaining items assess a respondent’s “current” status. In cognitive interview testing, patients completed the instrument in an average of 5.7 min (median of 5.0 min).

Consistent with current scientific best practices [17, 18], the selected recall period was based on qualitative concept elicitation data and confirmed as appropriate by patients during cognitive interviews. The current instrument is suggested for use in patients with CF aged ≥12 years. Although all patients, including those aged > 6 years, provided input on concepts, the readability (reading level) and terminology of CF-IQ has not yet been evaluated and adapted for this age group; thus, it is not advised for use with children aged 6–11 years. Following the qualitative instrument development steps described herein, efforts are underway to conduct a quantitative evaluation of the instrument’s psychometric and measurement properties.

## Discussion

Evaluating the merits of new drug therapies and other interventions for CF requires comprehensive and rigorous measurement of patients’ experiences alongside clinical endpoints. Existing HRQoL assessments in CF include observations of signs and symptoms by clinicians, qualitative symptom scales devised by investigators, and CF symptom-reporting instruments. Available tools for CF are largely symptom-focused.

Recent therapeutic advancements in CF treatment have created a specific need for a sensitive PRO tool to quantify and assess broader changes in CF burden for use in natural history studies, observational studies, and in clinical practice. In response, a PRO instrument for assessment of CF-related life impacts, the CF-IQ was developed*.* This is the first PRO instrument designed to assess the day-to-day impact of CF, including patients receiving CFTR-modulator therapies. Developed in accordance with scientific best practice guidelines [16–18], the CF-IQ may both complement existing instruments and offer additional insights on the impact of CF among patients treated with CFTR modulators.

There are several key strengths of the development process for the CF-IQ. First, CF-IQ was developed through rigorous, qualitative methods leveraging direct input from patients and caregivers to support initial concept selection. With its development rooted in the collection of qualitative concept elicitation data to the point of concept saturation, the subsequent item generation process resulted in content that reflects patient-derived language to frame questions and provide response options that are understandable and meaningful to patients. Second, elements of the CF-IQ’s distinct measurement approach, such as use of a shorter (7-day) recall period and greater use of severity-focused items, arose directly from patient interview findings and may provide added sensitivity to the instrument’s measurement profile when contrasted with the longer (two-week) recall period and use of frequency-focused item construction of the other PRO instuments.

In addition, the sample of patients involved in the CF-IQ development process reflects experiences with currently available treatment modalities, including the availability of newer CFTR modulator-based therapies. Thus, the instrument includes item content tailored to reflect the experiences of current patients consistent with published literature, as well as expanded coverage of issues experienced by patients managing CF with expanded therapeutic options. The expectation of extended survival for patients with CF using new and emerging therapies has informed the development of the CF-IQ. Greater emphasis on the future and on patients’ ability to exert control over the direction of their lives were key themes that emerged in qualitative interviews. Elements focused on future outlook are included in multiple domains designed to measure the burden of CF in terms of missed opportunities for social, work, and leisure activities, rather than solely via physical limitations.

In the initial concept elicitation interviews, many patients reported relatively low levels of perceived symptom burden, but when asked to provide detailed descriptions of day-to-day life experiences, patients reported substantial impacts such as high treatment burden and substantial time spent every day to manage their disease; limitations in social, personal, and work or school life due to CF; effect on family relationships; constant concern and stress about catching infections or being hospitalized; and restraint of themselves from thinking about future goals. This may reflect that patients who are accustomed to living with CF may state that they are feeling well; however, a more detailed description of their lives may illustrate a deeper, more pronounced impact of CF. For example, patients in the present sample describe engaging in activities rather than avoiding them, but they experience limitations such as needing to rest, taking breaks, or requiring assistance from others while engaged in the activity. The CF-IQ has incorporated this pattern of patient experience, for example, within the “stamina” domain, whereby the number of resting periods required to complete tasks is accounted for directly in the item scoring. Similarly, the CF-IQ includes items assessing frequency with which patients need to cancel plans due to changes in condition and assistance required from others. Inclusion of these perspectives has informed the development of items and conceptual domains of the CF-IQ to capture the overall burden of CF. Although the CF-IQ includes some concepts assessed in other existing instruments, it also incorporates important additions of patient-perceived impacts and offers expanded coverage of concepts related to future outlook, sense of control in managing one’s condition, relationships with others, activity limitations, and the burden of treatment compared to those included in previously-developed CF-specific instruments.

It is important to note study limitations. Although the size of the concept elicitation sample (*n* = 42 patients) was consistent with commonly used qualitative methods and comparable with those employed in development of other de novo PRO instruments [29, 30], there is a possibility for selection bias due to the nature of non-random sampling procedures. In-person interviews may have biased the sample against sicker patients who may not have been healthy enough to participate, although recruitment quotas were used within the purposive sampling approach to ensure representation of key patient characteristics routinely present in CF clinical trial samples (e.g. range of age, sex, disease severity, treatment type). Patient recruitment was also primarily conducted at major academic medical centers. Some of the interviewed patients received their care in smaller community practice settings, but the nature of the recruitment approach may have biased the sample to reflect the experience of patients with CF in larger practice settings.

## Conclusion

This qualitative study demonstrates initial content validity of the CF-IQ as a standardized PRO assessment tool for evaluating the impacts of CF, extending the scope of previous instruments to more sensitively quantify the burden of CF in patients treated with CFTR modulators and, thus, may offer a broader assessment of the efficacy of emerging therapies. Further evaluation of the instrument’s measurement properties is underway in current studies, which will be used to complete the psychometric validation of the instrument and may be used to reduce the number of items for some domains.

The CF-IQ can complement and extend information provided by symptom assessment, healthcare resource utilization, and existing PRO data to comprehensively quantify the burden of CF, and can be used in real-world studies to measure changes in the impact of CF.

## Data Availability

Specific data points can be made available upon request, due to the complex qualitative nature of the questionnaire and de-identified patient information.

## References

[1] Cystic Fibrosis Foundation. About cystic fibrosis. http://www.cff.org/AboutCF/. Accessed 8 Oct 2019.

[2] Cystic Fibrosis Foundation. Cystic Fibrosis Foundation Patient Registry: 2017 annual data report. Published in 2018. https://www.cff.org/Research/Researcher-Resources/Patient-Registry/2017-Patient-Registry-Annual-Data-Report.pdf. Accessed 8 Oct 2019.

[3] Sheppard MN, Nicholson AG (2002). The pathology of cystic fibrosis. Curr Diagn Pathol.

[4] O'Sullivan BP, Freedman SD (2009). Cystic fibrosis. Lancet.

[5] Farrell, P. M., et al. (2008). Guidelines for diagnosis of cystic fibrosis in newborns through older adults: Cystic Fibrosis Foundation consensus report. *J Pediatr, 153*, S4–S14. 10.1016/j.jpeds.2008.05.005 PMC2810958 18639722

[6] Abbott, J., et al. (2015). Longitudinal impact of demographic and clinical variables on health-related quality of life in cystic fibrosis. *BMJ Open, 5*, e007418. 10.1136/bmjopen-2014-007418 PMC4442198 25991453

[7] Dill EJ, Dawson R, Sellers DE, Robinson WM, Sawicki GS (2013). Longitudinal trends in health-related quality of life in adults with cystic fibrosis. Chest.

[8] Sawicki GS, Tiddens H (2012). Managing treatment complexity in cystic fibrosis: Challenges and opportunities. Pediatr Pulmonol.

[9] Sawicki GS, Sellers DE, Robinson WM (2009). High treatment burden in adults with cystic fibrosis: Challenges to disease self-management. J Cyst Fibros.

[10] Jamieson N, Fitzgerald D, Singh-Grewal D, Hanson CS, Craig JC, Tong A (2014). Children’s experiences of cystic fibrosis: A systematic review of qualitative studies. Pediatrics.

[11] Hassan M, Bonafede MM, Limone B, Hodgkins P, Sawicki G (2016). The burden of cystic fibrosis: Pulmonary exacerbations and health care resource utilization in a commercially insured population in the United States. Value Health.

[12] Ratjen F, Tullis E, Albert RK, Spiro SG, Jett JR (2008). Cystic fibrosis. Clinical respiratory medicine.

[13] McCoy KS, Quittner AL, Oermann CM, Gibson RL, Retsch-Bogart GZ, Montgomery AB (2008). Inhaled aztreonam lysine for chronic airway Pseudomonas aeruginosa in cystic fibrosis. Am J Respir Crit Care Med.

[14] Donaldson SH, Bennett WD, Zeman KL, Knowles MR, Tarran R, Boucher RC (2006). Mucus clearance and lung function in cystic fibrosis with hypertonic saline. N Engl J Med.

[15] Mogayzel Jr., P. J., et al. (2103). Cystic fibrosis pulmonary guidelines. Chronic medications for maintenance of lung health. *Am J Respir Crit Care Med, 187*, 680–689. 10.1164/rccm.201207-1160oe 23540878

[16] US Department of Health and Human Services, US FDA Center for Drug Evaluation and Research; US FDA Center for Biologics Evaluation and Research; US FDA Center for Devices and Radiological Health. (2009). Guidance for industry: patient-reported outcome measures: use in medical product development to support labeling claims. https://www.fda.gov/media/77832/download. Accessed 9 Apr 2020.

[17] Patrick, D. L., et al. (2011). Content validity—Establishing and reporting the evidence in newly developed patient-reported outcomes (PRO) instruments for medical product evaluation: ISPOR PRO good research practices task force report: Part 1—Eliciting concepts for a new PRO instrument. *Value Health, 14*, 967–977. 10.1016/j.jval.2011.06.014 22152165

[18] Patrick, D. L., et al. (2011). Content validity—Establishing and reporting the evidence in newly developed patient-reported outcomes (PRO) instruments for medical product evaluation: ISPOR PRO good research practices task force report: Part 2—Assessing respondent understanding. *Value Health, 14*, 978–988. 10.1016/j.jval.2011.06.013 22152166

[19] Gee L, Abbott J, Conway SP, Etherington C, Webb AK (2000). Development of a disease specific health related quality of life measure for adults and adolescents with cystic fibrosis. Thorax.

[20] Quittner, A. L., et al. (2000). Translation and linguistic validation of a disease-specific quality of life measure for cystic fibrosis. *J Pediatr Psychol, 25*, 403–414. 10.1093/jpepsy/25.6.403 10980045

[21] Quittner AL, Buu A, Messer MA, Modi AC, Watrous M (2005). Development and validation of the cystic fibrosis questionnaire in the United States; a health-related quality-of-life measure for cystic fibrosis. Chest.

[22] Quittner, A. L., et al. (2012). Psychometric evaluation of the cystic fibrosis questionnaire-revised in a national sample. *Qual Life Res, 21*, 1279–1290. 10.1007/s11136-011-0091-5 22240933

[23] Goldbeck L, Schmitz TG, Henrich G, Herschbach P (2003). Questions on life satisfaction for adolescents and adults with cystic fibrosis: Development of a disease-specific questionnaire. Chest.

[24] Saiman, L., et al. (2003). Azithromycin in patients with cystic fibrosis chronically infected with *Pseudomonas aeruginosa*. *JAMA, 290*, 1749–1756. 10.1001/jama.290.13.1749 14519709

[25] Modi AC, Lim CS, Driscoll KA, Piazza-Waggoner C, Quittner AL, Wooldrigde J (2010). Changes in pediatric health-related quality of life in cystic fibrosis after IV antibiotic treatment for pulmonary exacerbations. J Clin Psychol Med Settings.

[26] Quittner, A., et al. (2015). Effect of ivacaftor treatment in patients with cystic fibrosis and the G551D-CFTR mutation: Patient-reported outcomes in the STRIVE randomized, controlled trial. *Health Qual Life Outcomes, 13*, 93. 10.1186/s12955-015-0293-6 PMC4702321 26135562

[27] Muhr T (2004). User's manual for ATLAS.Ti 5.0.

[28] Beatty PC, Willis GB (2007). Research synthesis: The practice of cognitive interviewing. Public Opin Q.

[29] McCarrier KP, Atkinson TM, DeBusk KP, Liepa AM, Scanlon M, Coons SJ (2016). Qualitative development and content validity of the non-small cell lung Cancer symptom assessment questionnaire (NSCLC-SAQ), a patient-reported outcome instrument. Clin Ther.

[30] Goss CH, Edwards TC, Ramsey BW, Aitken ML, Patrick DL (2009). Patient-reported respiratory symptoms in cystic fibrosis. J Cyst Fibros.

